# Evaluation of the Relationship Between the Pan-Immune-Inflammation Score and the Systemic Immune-Inflammation Index and Hypertension: A Retrospective Cross-Sectional Study

**DOI:** 10.3390/jcm15113996

**Published:** 2026-05-22

**Authors:** Safiye Kübra Çetindağ Karatlı, Ebru Uğraş, Erhan Şimşek, Ahmet Keskin

**Affiliations:** 1 Department of Family Medicine, Ankara Yıldırım Beyazıt University, Ankara 06760, Türkiye; 2 Department of Family Medicine, Ankara Bilkent City Hospital, Ankara 06760, Türkiye

**Keywords:** hypertension, inflammation, pan-immune-inflammation value, systemic immune-inflammation index

## Abstract

**Background:** Inflammation is believed to play a significant role in the pathophysiology of hypertension (HT). The aim of this study was to evaluate the relationship between the systemic immune-inflammation index (SII) and the pan-immune-inflammation value (PIV) and HT in adults. **Methods:** A total of 1060 adult individuals who presented between 1 December 2025 and 1 February 2026, were included. Participants were grouped according to the presence of HT. SII and PIV were calculated. Univariate and multivariate logistic regression analyses were performed to identify factors independently associated with HT. Due to the high correlation between SII and PIV, they were evaluated in separate models. Discriminatory performance was examined using ROC analysis, and correlations were assessed using the Spearman test. **Results:** HT was present in 18.1% of participants. In the HT group, SII and PIVs were significantly higher (*p* < 0.001 for both). In univariate analysis, older age (*p* < 0.001), male gender (*p* = 0.030), presence of comorbidities (*p* < 0.001), high SII (*p* < 0.001), and high PIV (*p* < 0.001) were found to be associated with HT. In multivariate analyses, age and comorbidities remained independent predictors in both models (all *p* < 0.001). In the multivariable models adjusted for available covariates, high SII was significantly associated with HT in the SII model (adjusted OR: 2.035; 95% CI: 1.374–3.010; *p* < 0.001). Similarly, high PIV was significantly associated with HT in the PIV model (adjusted OR: 5.577; 95% CI: 3.398–9.160; *p* < 0.001). In the ROC analysis, both indices demonstrated modest predictive ability, with PIV showing slightly higher performance compared to SII (AUC: 0.648 vs. 0.623). A positive correlation was observed between the duration of HT and both SII (r = 0.700; *p* < 0.001) and PIV (r = 0.847; *p* < 0.001). **Conclusions:** The finding that SII and PIV were significantly associated with HT after adjustment for available covariates supports the potential role of systemic inflammation in the pathophysiology of HT. These indices, which can be easily calculated from routine laboratory parameters and do not require additional costs, may potentially assist in risk assessment in clinical practice.

## 1. Introduction

Hypertension (HT) is a major global public health issue and one of the leading modifiable risk factors for cardiovascular disease, stroke, and chronic kidney disease. According to the World Health Organization, approximately 1.28 billion adults aged 30–79 years worldwide have hypertension, corresponding to a global prevalence of nearly 32% [1,2]. In Türkiye, the prevalence of hypertension in adults over 18 years of age is reported to be around 30–35% [3]. Despite advances in diagnosis and management, a substantial proportion of individuals with hypertension remain undiagnosed or inadequately controlled, leading to significant cardiovascular morbidity and mortality. The pathophysiology of hypertension is complex and multifactorial. In addition to classical mechanisms such as sympathetic nervous system overactivity, renin–angiotensin–aldosterone system (RAAS) activation, and impaired renal sodium excretion, chronic low-grade systemic inflammation has been increasingly recognized as a significant contributor in recent years [4,5,6]. Inflammation promotes the development and progression of hypertension through several interrelated pathways, including endothelial dysfunction, increased oxidative stress, vascular remodeling, arterial stiffness, and sodium retention. In this process, neutrophils contribute to oxidative stress and endothelial injury, monocytes facilitate inflammatory cytokine production and vascular wall infiltration, while lymphocytes—particularly regulatory T cells—play important modulatory and anti-inflammatory roles [4,5,6,7].

In recent years, easily calculable hematological indices derived from routine complete blood count parameters have emerged as practical and cost-effective markers of systemic inflammation. Among these, the Systemic Immune-Inflammation Index (SII), calculated as (Neutrophil × Platelet)/Lymphocyte, and the Pan-Immune-Inflammation Value (PIV), calculated as (Neutrophil × Platelet × Monocyte)/Lymphocyte, are considered two of the most promising indices. These composite markers reflect the delicate balance between pro-inflammatory cells (neutrophils, platelets, and monocytes) and anti-inflammatory cells (lymphocytes). Previous studies have shown that elevated SII and PIV levels are associated with poor clinical outcomes in various cardiovascular diseases, malignancies, and other inflammatory conditions [8,9,10]. More recently, growing evidence has indicated positive associations between these indices and both the presence and severity of hypertension [9,11]. However, direct head-to-head comparative studies evaluating SII and PIV in the same population are still limited.

Therefore, the aim of this retrospective cross-sectional study was to evaluate the associations of SII and PIV with hypertension in adult participants and to compare their discriminatory performance in predicting hypertension.

## 2. Materials and Methods

### 2.1. Study Design and Participant Population

This retrospective, single-center study included participants aged 18 years and older who presented to the Family Medicine outpatient clinic at Ankara Bilkent City Hospital between 1 December 2025 and 1 February 2026. Participants were divided into two groups based on the presence or absence of a diagnosis of HT. The diagnosis of HT was based on the relevant ICD codes recorded in the hospital’s electronic medical record system. In this study, participants were not classified according to any other disease group. Comorbidity status was evaluated as a composite variable including diabetes mellitus, coronary artery disease, hyperlipidemia, and other cardiometabolic conditions identified from the electronic medical record system.

**Figure 1:**
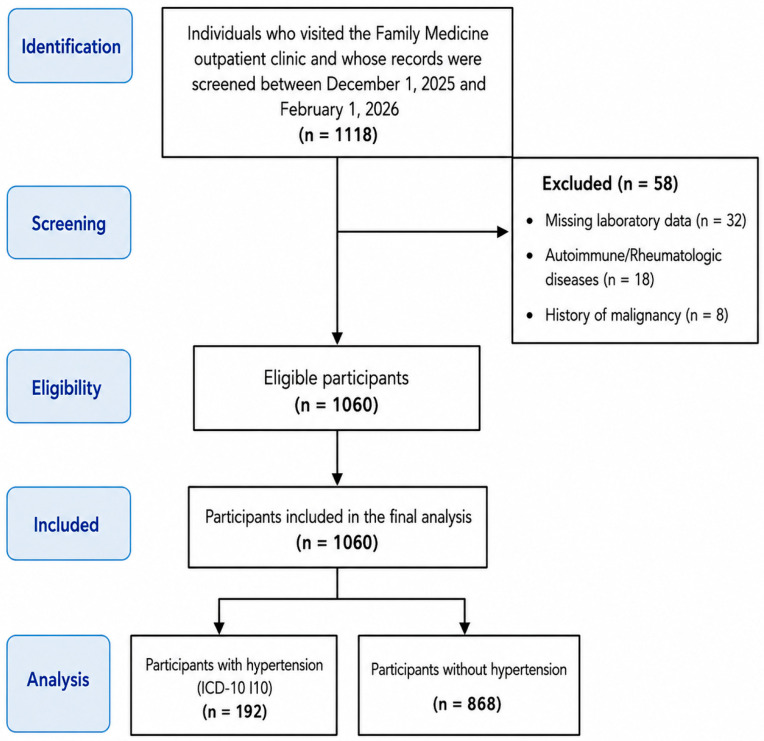
STROBE flow diagram of the study participants.

A flow diagram of the study participant selection process was constructed according to the STROBE (Strengthening the Reporting of Observational Studies in Epidemiology) guidelines (Figure 1). A total of 1118 participants were initially screened from the hospital database between 1 December 2025 and 1 February 2026. After applying inclusion and exclusion criteria, 1060 participants with complete data were included in the final analysis. Participants were excluded because of missing laboratory parameters (*n* = 58) or predefined exclusion criteria, including acute infection, malignancy, and autoimmune or rheumatologic disease.

#### 2.1.1. Inclusion Criteria

- Participants aged 18 years and older;
- Participants with a clearly documented HT status according to ICD codes in the hospital electronic medical record system;
- Participants with complete blood count parameters available (neutrophils, lymphocytes, monocytes, and platelets) necessary for the calculation of SII and PIV;
- Participants with available demographic and clinical data in the hospital records;
- Participants who presented to the Family Medicine outpatient clinic between 1 December 2025 and 1 February 2026.

Although the study included participants aged 18 years and older, the median age of the overall study population was 40 years (IQR: 22). Age was presented as a continuous variable in descriptive analyses and was dichotomized according to the median value of the study population for regression analyses.

#### 2.1.2. Exclusion Criteria

To ensure the homogeneity of the study population and to eliminate conditions that could affect inflammatory markers, the following criteria were defined as exclusion criteria:

- Presence of acute infection (evaluated by excluding participants with clinical symptoms consistent with infection at presentation);
- Chronic infectious diseases;
- Autoimmune diseases;
- Rheumatologic diseases;
- History of malignancy;
- Missing laboratory data.

In accordance with these criteria, a total of 1060 participants who met the eligibility criteria were included in the study.

No formal sample size calculation was performed prior to the study, as this was a retrospective study based on all eligible participants who presented to the Family Medicine outpatient clinic during the predefined 2-month period from 1 December 2025 to 1 February 2026) and met the inclusion/exclusion criteria. A total of 1060 participants who fulfilled the eligibility criteria were included. This sample size was considered sufficient given the relatively high prevalence of HT in the Turkish adult population (~30%) and the aim of the study to detect associations between inflammatory indices and HT.

### 2.2. Data Collection and Definitions

Study data were retrospectively obtained from the hospital’s electronic medical record system. Demographic characteristics (age and sex), HT status, comorbidities, and hematological parameters were extracted by researchers. HT was defined according to the presence of ICD-10 code I10 recorded in the electronic medical record system by the treating physicians, while comorbidities were identified using relevant ICD codes. Laboratory measurements corresponded to the outpatient admission period and were considered to reflect the participants’ clinical status.

Hematological parameters, including hemoglobin, neutrophil, lymphocyte, monocyte, and platelet counts, were obtained from routine complete blood count analyses performed in the hospital’s central laboratory. Systemic inflammatory status was assessed using the systemic immune-inflammation index (SII) and pan-immune-inflammation value (PIV), which were calculated by the investigators according to the following formulas:

SII = (Neutrophil × Platelet)/Lymphocyte

PIV = (Neutrophil × Platelet × Monocyte)/Lymphocyte

All hematological parameters were expressed in ×10^9^/L units. Data extraction and index calculations were independently cross-checked by the researchers to ensure accuracy.

All extracted data underwent quality control procedures. Laboratory values outside the physiological range were reviewed for possible entry errors by cross-checking with the original laboratory reports and the hospital laboratory information system. Complete-case analysis was performed for missing data. Participants with missing values in key variables required for SII and PIV calculation (neutrophil, lymphocyte, monocyte, or platelet counts) or missing essential demographic/clinical information were excluded from the study according to the predefined exclusion criteria. No imputation methods were applied, as only participants with complete laboratory and clinical records were included to ensure data reliability and minimize potential bias.

### 2.3. Statistical Analysis

Statistical analyses were performed using IBM SPSS Statistics (version 25.0). The distribution of continuous variables was assessed using visual and analytical methods. Since the variables were not normally distributed, they were expressed as median, interquartile range, and minimum–maximum values. The Mann–Whitney U test was used for comparisons between groups, and the chi-square test was used for categorical variables. Univariate and multivariate logistic regression analyses were conducted to identify factors associated with HT after adjustment for available covariates. The predictive performance of SII and PIV for HT was evaluated using receiver operating characteristic (ROC) curve analysis. Optimal cut-off values were determined using the Youden index, and based on these values, variables were categorized into binary groups (low/high) and included in logistic regression analyses. For regression analyses, age was dichotomized according to the median age of the study population in order to facilitate categorical risk comparison between groups. Due to the high correlation between SII and PIV, separate models were constructed to avoid multicollinearity. A two-tailed *p* value of <0.05 was considered statistically significant in all analyses.

### 2.4. Ethical Approval

The study was approved by the Ethics Committee of Ankara Bilkent City Hospital with decision number 1933 dated 4 February 2026 and was conducted in accordance with the principles of the Declaration of Helsinki.

## 3. Results

A total of 1060 participants were included in the study, of whom 192 (18.1%) had HT. Participants with HT were significantly older compared to those without HT (median: 53.5 [IQR: 26] years [25–91] vs. 33.0 [IQR: 20] years [18–91]; *p* < 0.001). The median age of the overall study population was 40 years. When gender distribution was examined, the proportion of males was higher in the HT group (80/192 [41.7%] vs. 290/868 [33.4%]; *p* = 0.030). The presence of comorbidities was markedly higher in the HT group (144/192 [75.0%] vs. 178/868 [20.6%]; *p* < 0.001). The proportion of participants in the older age group was also significantly higher in the HT group (154/192 [80.2%] vs. 412/868 [47.5%]; *p* < 0.001) (Table 1).

When laboratory parameters were evaluated, neutrophil (median 4.59 vs. 3.83 × 10^9^/L; *p* < 0.001) and monocyte (0.61 vs. 0.56 × 10^9^/L; *p* = 0.002) levels were significantly higher in the HT group, whereas lymphocyte levels were lower (2.11 vs. 2.42 ×10^9^/L; *p* < 0.001). There was no significant difference in hemoglobin and platelet values (*p* = 0.213 and *p* = 0.669, respectively). Both SII and PIVs were significantly higher in the HT group (SII: 541.51 vs. 440.95; PIV: 343.00 vs. 240.04; *p* < 0.001 for both) (Table 2).

In the univariate logistic regression analysis, older age group (OR: 4.485; 95% CI: 3.070–6.554; *p* < 0.001), male sex (OR: 1.424; 95% CI: 1.034–1.960; *p* = 0.030), presence of comorbidity (OR: 11.596; 95% CI: 8.042–16.718; *p* < 0.001), high SII (OR: 2.149; 95% CI: 1.525–3.028; *p* < 0.001), and high PIV (OR: 3.979; 95% CI: 2.636–6.005; *p* < 0.001) were found to be significantly associated with HT (Table 3).

Multivariate logistic regression analyses were performed as two separate models to avoid potential collinearity due to the strong correlation observed between SII and PIV. Older age group (SII model: adjusted OR: 2.036; 95% CI: 1.370–3.025; *p* < 0.001; PIV model: adjusted OR: 2.278; 95% CI: 1.512–3.430; *p* < 0.001) and presence of comorbidity (SII model: adjusted OR: 6.669; 95% CI: 4.639–9.579; *p* < 0.001; PIV model: adjusted OR: 7.240; 95% CI: 4.981–10.531; *p* < 0.001) remained independent predictors in both models. In the SII model, high SII was significantly associated with HT after adjustment for available covariates (adjusted OR: 2.035; 95% CI: 1.374–3.010; *p* < 0.001). Similarly, in the PIV model, high PIV was significantly associated with HT after adjustment for available covariates (adjusted OR: 5.577; 95% CI: 3.398–9.160; *p* < 0.001). Male sex did not show independent significance in either model (SII model: adjusted OR: 1.342; 95% CI: 0.941–1.913; *p* = 0.103; PIV model: adjusted OR: 1.337; 95% CI: 0.930–1.921; *p* = 0.116) (Table 4 and Table 5).

In the Spearman correlation analysis performed in 192 participants with available HT duration data, a strong positive correlation was found between HT duration and SII (r = 0.700; *p* < 0.001), and an even stronger correlation was observed between HT duration and PIV (r = 0.847; *p* < 0.001). A very strong correlation was also detected between SII and PIV (r = 0.862; *p* < 0.001) (Table 6).

**Figure 2:**
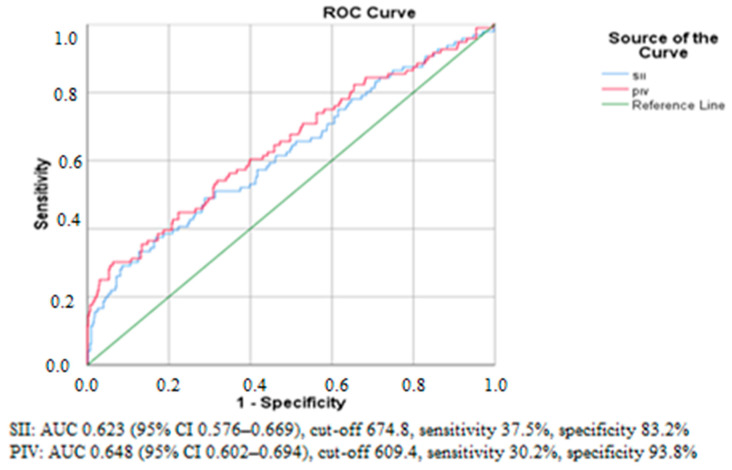
ROC curve analysis of SII and PIV for predicting hypertension.

In the ROC curve analysis, the predictive performance of SII for HT was found to be AUC = 0.623 (95% CI: 0.576–0.669; *p* < 0.001), while the AUC for PIV was 0.648 (95% CI: 0.602–0.694; *p* < 0.001). Although PIV showed slightly better performance compared to SII, the discriminative ability of both indices was modest. Optimal cut-off values were determined using the Youden index as 674.8 for SII and 609.4 for PIV (Figure 2, Table 7).

## 4. Discussion

This study demonstrated that both PIV and SII were significantly associated with HT in multivariable models adjusted for available covariates. Notably, PIV exhibited a stronger association than SII. These findings support the growing body of evidence linking systemic inflammation to the pathophysiology of HT.

Indices reflecting both inflammation and nutritional status, such as HALP (Hemoglobin, albumin, lymphocyte, platelet), PNI (Prognostic Nutritional Index), and AGR (Albumin-to-Globulin Ratio), have increasingly been investigated for their prognostic and predictive value in various clinical conditions [11,12,13,14,15]. Similarly, combined inflammatory indices such as PIV and SII have been reported to show significant associations in various clinical settings, particularly in cardiovascular diseases [10,11,16,17]. These findings suggest that these indices are not specific to a single disease but may serve as common biomarkers across different pathophysiological processes.

Inflammation contributes to the development and progression of hypertension through multiple mechanisms, including endothelial dysfunction, oxidative stress, vascular remodeling, and activation of the renin–angiotensin–aldosterone system [18]. In addition, chronic inflammatory burden has been associated with increased blood pressure variability and target organ damage [16,17]. In the current study, both SII and PIV demonstrated positive correlations with HT duration, further supporting the potential relationship between chronic inflammation and long-term disease burden.

Our findings are consistent with the existing literature supporting the association between systemic inflammation and HT. Studies based on National Health and Nutrition Examination Survey (NHANES) data have shown that elevated PIV levels are associated with adverse clinical outcomes, including increased mortality, among hypertensive individuals [19]. Similarly, higher SII levels have been independently associated with both the prevalence and risk of HT [20,21]. In the present study, PIV demonstrated slightly better discriminatory performance than SII in ROC analysis; however, both indices showed only modest predictive ability overall. The relatively high specificity but low sensitivity observed for both markers suggests that PIV and SII should not be considered standalone screening or diagnostic tools for HT, but rather complementary inflammatory biomarkers. Furthermore, the positive correlations observed between HT duration and both PIV and SII support the concept that inflammatory burden may be associated not only with the presence of HT but also with disease persistence and long-term clinical burden.

The main strengths of this study include its relatively large sample size and the combined evaluation of two easily calculable inflammatory indices, SII and PIV. These indices are derived from routine complete blood count parameters, are cost-effective, and may be readily applicable in clinical practice. In addition, the use of separate regression models to avoid potential collinearity and the comparison of discriminatory performance using ROC analysis further strengthen the methodological design of the study.

This study has several limitations. First, the retrospective cross-sectional design precludes causal inference. Second, due to reliance on routine electronic medical records, detailed medication history—including antihypertensive agents, statins, aspirin, and other anti-inflammatory drugs—was not consistently available. These medications are known to influence hematological parameters and systemic inflammatory indices such as SII and PIV. Consequently, we could not adjust for their effects, and the absence of medication data represents an important source of potential residual confounding. Third, comorbidity was assessed as a composite variable encompassing diabetes mellitus, coronary artery disease, hyperlipidemia, and other cardiometabolic conditions. Due to the retrospective nature of the study, limited sample sizes in certain subgroups, and to avoid overfitting in multivariable models, individual comorbidities were not analyzed separately. This approach may have resulted in residual confounding. Fourth, anthropometric measurements such as body mass index and waist circumference were not consistently recorded. Given the well-established relationship between obesity, inflammation, and HT, the absence of these variables is an important limitation. Finally, age was analyzed as a categorical variable based on the median value of the study population rather than as a continuous variable. Although this approach improved interpretability and facilitated categorical risk comparison in the regression models, it may have limited a more detailed evaluation of age-related risk profiles and potential nonlinear associations with HT. Additionally, as the study was conducted in a single-center family medicine outpatient clinic, selection bias may exist. Participants primarily presented for routine check-ups, follow-up of chronic conditions, or minor complaints. Therefore, the findings may not be fully generalizable to the broader adult population, particularly to individuals who do not regularly utilize primary healthcare services.

Despite these limitations, the relatively large sample size and the utilization of easily accessible, cost-free routine laboratory parameters remain important strengths of this study. Future prospective cohort and interventional studies that comprehensively adjust for medication use, detailed comorbidity profiles, anthropometric measurements, and traditional inflammatory markers are needed to validate our findings and clarify the clinical utility of SII and PIV in HT management.

## 5. Conclusions

In conclusion, SII and PIV levels were significantly higher in participants with HT than in those without, supporting a potential link between systemic inflammation and HT. Both indices were significantly associated with HT in multivariable models adjusted for available covariates; however, PIV showed slightly better discriminative performance than SII, although the overall predictive ability of both biomarkers remained modest.

The relatively high specificity but low sensitivity of SII and PIV indicate that these indices may be more useful as supportive inflammatory biomarkers rather than standalone screening or diagnostic tools. Given that they can be easily calculated from routine complete blood count parameters at no additional cost, they may serve as complementary tools for assessing inflammatory burden in hypertensive individuals, particularly in primary care settings in Türkiye and similar healthcare systems. Their utility may be more limited in rural or low-resource settings with restricted laboratory access.

Nevertheless, due to the retrospective and cross-sectional design of the study and potential uncontrolled confounders, the findings should be interpreted cautiously. Future prospective cohort studies are needed to validate these results.

## Figures and Tables

**Table 1:** Baseline demographic and clinical characteristics according to HT status.

Variable	No HT (*n* = 868)	HT (*n* = 192)	*p* Value
Age (years)	33.0 (20) [18–91]	53.5 (26) [25–91]	<0.001
Female, *n* (%)	578 (66.6)	112 (58.3)	0.03
Male, *n* (%)	290 (33.4)	80 (41.7)	
Comorbidity present, *n* (%)	178 (20.6)	144 (75.0)	<0.001
High age group, *n* (%)	412 (47.5)	154 (80.2)	<0.001

Footnote: Data are presented as median (interquartile range) [minimum–maximum] or number (%). Comparisons were performed using the Mann–Whitney U test or chi-square test. High age group was defined according to the median age of the study population (40 years). Abbreviations: HT: Hypertension.

**Table 2:** Laboratory parameters and inflammatory indices according to HT status.

Variable	No HT (*n* = 868)	HT (*n* = 192)	*p* Value
Hemoglobin (g/dL)	13.9 (2.1) [8.1–18.3]	13.8 (2.9) [9.5–18.1]	0.213
Neutrophils (×10^9^/L)	3.83 (1.75) [0.80–10.32]	4.59 (2.75) [1.09–15.43]	<0.001
Monocytes (×10^9^/L)	0.56 (0.23) [0.00–1.68]	0.61 (0.29) [0.17–8.21]	0.002
Lymphocytes (×10^9^/L)	2.42 (0.92) [0.11–7.60]	2.11 (1.28) [0.16–4.97]	<0.001
Platelets (×10^9^/L)	267.0 (83.0) [105–589]	264.5 (99.75) [52–467]	0.669
SII	440.95 (277.91) [8.90–7958.45]	541.51 (481.68) [69.98–7233.48]	<0.001
PIV	240.04 (217.15) [0–1184.86]	343.00 (536.24) [11.90–5714.45]	<0.001

Footnote: All continuous variables were non-normally distributed and are presented as median (IQR) [min–max]. Abbreviations: HT: Hypertension; SII: Systemic Immune-Inflammation Index; PIV: Pan-Immune-Inflammation Value.

**Table 3:** Univariable logistic regression analysis for factors associated with HT.

Variable	OR	95% CI	*p* Value
High age group	4.485	3.070–6.554	<0.001
Male sex	1.424	1.034–1.960	0.03
Presence of comorbidity	11.596	8.042–16.718	<0.001
High SII	2.149	1.525–3.028	<0.001
High PIV	3.979	2.636–6.005	<0.001

Footnote: Hypertension status was used as the dependent variable. High age group was defined according to the median age of the study population (40 years). Abbreviations: HT: Hypertension; SII: Systemic Immune-Inflammation Index; PIV: Pan-Immune-Inflammation Value.

**Table 4:** Multivariable logistic regression analysis for factors associated with hypertension (SII model).

Variable	Adjusted OR	95% CI	*p* Value
Male sex	1.342	0.941–1.913	0.103
High age group	2.036	1.370–3.025	<0.001
Presence of comorbidity	6.669	4.639–9.579	<0.001
High SII	2.035	1.374–3.010	<0.001

Footnote: Variables entered into the model were sex, age group, presence of comorbidity, and SII group. High age group was defined according to the median age of the study population (40 years). Abbreviations: HT: Hypertension; SII: Systemic Immune-Inflammation Index.

**Table 5:** Multivariable logistic regression analysis for factors associated with hypertension (PIV model).

Variable	Adjusted OR	95% CI	*p* Value
Male sex	1.337	0.930–1.921	0.116
High age group	2.278	1.512–3.430	<0.001
Presence of comorbidity	7.24	4.981–10.531	<0.001
High PIV	5.577	3.398–9.160	<0.001

Footnote: Variables entered into the model were sex, age group, presence of comorbidity, and PIV group. High age group was defined according to the median age of the study population (40 years). Abbreviations: HT: Hypertension; PIV: Pan-Immune-Inflammation Value.

**Table 6:** Spearman correlation analysis between hypertension duration, SII, and PIV.

Variables	HT Duration	SII	PIV
HT duration	1	0.7	0.847
SII	0.7	1	0.862
PIV	0.847	0.862	1

Footnote: Spearman correlation analysis was performed. PIV and SII were both significantly correlated with hypertension duration (*p* < 0.001). *n* = 192 for hypertension duration correlations; *n* = 1060 for SII–PIV correlation. Correlation is significant at the 0.01 level (two-tailed). Abbreviations: HT: Hypertension; SII: Systemic Immune-Inflammation Index; PIV: Pan-Immune-Inflammation Value.

**Table 7:** ROC Analysis and Diagnostic Performance of SII and PIV in Predicting Hypertension.

Parameter	AUC (95% CI)	Cut-Off	Sensitivity	Specificity	PPV	NPV
SII	0.623 (0.576–0.669)	674.8	37.50%	83.20%	33.00%	85.70%
PIV	0.648 (0.602–0.694)	609.4	30.20%	93.80%	51.80%	85.90%

Abbreviations: AUC, area under the curve; CI, confidence interval; SII, systemic immune-inflammation index; PIV, pan-immune-inflammation value; PPV, positive predictive value; NPV, negative predictive value.

## Data Availability

The datasets generated and analyzed during the current study are available from the corresponding author upon reasonable request.

## Abbreviations

The following abbreviations are used in this manuscript:

HT	Hypertension
SII	Systemic immune-inflammation index
PIV	Pan-immune-inflammation value
HALP	Hemoglobin, albumin, lymphocyte, platelet
PNI	Prognostic nutritional index
AGR	Albumin-to-Globulin Ratio
NHANES	National Health and Nutrition Examination Survey

## References

[1] Jones D.W., Ferdinand K.C., Taler S.J., Johnson H.M., Shimbo D., Abdalla M., Altieri M.M., Bansal N., Bello N.A., Bress A.P. (2025). 2025 AHA/ACC/AANP/AAPA/ABC/ACCP/ACPM/AGS/AMA/ASPC/NMA/PCNA/SGIM Guideline for the Prevention, Detection, Evaluation and Management of High Blood Pressure in Adults: A Report of the American College of Cardiology/American Heart Association Joint Committee on Clinical Practice Guidelines. Circulation.

[2] World Health Organization (WHO) Hypertension. https://www.who.int/news-room/fact-sheets/detail/hypertension.

[3] Sengul S., Akpolat T., Erdem Y., Derici U., Arici M., Sindel S., Karatan O., Turgan C., Hasanoglu E., Caglar S. (2016). Changes in Hypertension Prevalence, Awareness, Treatment, and Control Rates in Turkey from 2003 to 2012. J. Hypertens..

[4] Harrison D.G., Guzik T.J., Lob H.E., Madhur M.S., Marvar P.J., Thabet S.R., Vinh A., Weyand C.M. (2011). Inflammation, Immunity, and Hypertension. Hypertension.

[5] Rodriguez-Iturbe B., Pons H., Johnson R.J. (2017). Role of the Immune System in Hypertension. Physiol. Rev..

[6] Guzik T.J., Nosalski R., Maffia P., Drummond G.R. (2024). Immune and Inflammatory Mechanisms in Hypertension. Nat. Rev. Cardiol..

[7] Akyar S., Dağdeviren H.N. (2026). Systemic Immune-Inflammation Index: A Novel Tool for Hypertension Management in Primary Care. BMC Prim. Care.

[8] Sun S., Fu J., Yang J., Zhao L., Zhao B., Zhou Y. (2025). Correlation Analysis of Systemic Immune Inflammation Index with the Occurrence and Clinical Outcomes of Hypertension: A Systematic Review and Meta-Analysis. Front. Cardiovasc. Med..

[9] Chen Y., Li Y., Liu M., Xu W., Tong S., Liu K. (2024). Association between Systemic Immunity-Inflammation Index and Hypertension in US Adults from NHANES 1999–2018. Sci. Rep..

[10] Ye Z., Hu T., Wang J., Xiao R., Liao X., Liu M., Sun Z. (2022). Systemic Immune-Inflammation Index as a Potential Biomarker of Cardiovascular Diseases: A Systematic Review and Meta-Analysis. Front. Cardiovasc. Med..

[11] Weeks C.J., Bekele B.B., Altvater M., Cheng J., Zhu H., Huang Y., Jehu D.A., Simon A.B., Li W., Dong Y. (2025). The Association between Short-Term Blood Pressure Variability and Inflammation in Healthy Young Adults. J. Cardiovasc. Dev. Dis..

[12] Karatlı S., Yazılıtaş D. (2025). Low HALP Score Predicts Prolonged Hospitalization in Solid Tumor Patients with Febrile Neutropenia. Curr. Oncol..

[13] Karatlı S., Yazılıtaş D., Kaya S., Baraklı E.Y., Çelik S., İmamoğlu G.İ. (2026). Prognostic Role of Nutritional and Inflammatory Indices in Predicting Adverse Clinical Outcomes in Unplanned Hospitalized Oncology Patients. J. Clin. Med..

[14] Aboukhater D., Morad B., Nasrallah N., Nasser S.A., Sahebkar A., Kobeissy F., Boudaka A., Eid A.H. (2023). Inflammation and Hypertension: Underlying Mechanisms and Emerging Understandings. J. Cell. Physiol..

[15] Wang Q., Zhong W., Xiao Y., Lin G., Lu J., Xu L., Zhang G., Liu A., Du J., Wu B. (2024). Pan-Immune-Inflammation Value Predicts Clinical Outcomes in Critically Ill Patients. Cancer Sci..

[16] Ma L.L., Xiao H.B., Zhang J., Liu Y.H., Hu L.K., Chen N., Chu X., Dong J., Yan Y.X. (2024). Association between Systemic Immune Inflammatory/Inflammatory Response Index and Hypertension: A Cohort Study of Functional Community. Nutr. Metab. Cardiovasc. Dis..

[17] Ateş M.S., Yıldırım A., Sökmen E. (2025). Association between Pan-Immune-Inflammation Value and Dipper/Non-Dipper Status in Newly Diagnosed Hypertensive Patients. J. Inflamm. Res..

[18] Guzik T.J., Touyz R.M. (2017). Oxidative Stress, Inflammation, and Vascular Aging in Hypertension. Hypertension.

[19] Wu B., Zhang C., Lin S., Zhang Y., Ding S., Song W. (2023). The Relationship between the Pan-Immune-Inflammation Value and Long-Term Prognoses in Patients with Hypertension: National Health and Nutrition Examination Study, 1999–2018. Front. Cardiovasc. Med..

[20] Jin N., Huang L., Hong J., Zhao X., Hu J., Wang S., Zhou L., Wang L., Zhang J., Lu Y. (2023). The Association between Systemic Inflammation Markers and the Prevalence of Hypertension. BMC Cardiovasc. Disord..

[21] Zhao Y., Shao W., Zhu Q., Zhang R., Sun T., Wang B., Hu X. (2023). Association between Systemic Immune-Inflammation Index and Metabolic Syndrome and Its Components: Results from the National Health and Nutrition Examination Survey 2011–2016. J. Transl. Med..

